# Supplementary material for: Tbx1 haploinsufficiency leads to local skull deformity, paraflocculus and flocculus dysplasia, and motor-learning deficit in 22q11.2 deletion syndrome
Source: Nat Commun. 2024 Dec 5;15:10510. doi: 10.1038/s41467-024-54837-3 (PMC11621701; doi:10.1038/s41467-024-54837-3)
Supplement: Supplementary file 1 — Supplementary Information [file 41467_2024_54837_MOESM1_ESM.pdf]

# Supplementary information

# *Tbx1* haploinsufficiency leads to local skull deformity, paraflocculus and flocculus dysplasia, and motor-learning deficit in 22q11.2 deletion syndrome

Tae-Yeon Eom[1], J. Eric Schmitt[2,3], Yiran Li[1], Christopher M. Davenport[1], Jeffrey Steinberg[4], Audrey Bonnan[5], Shahinur Alam[1,6], Young Sang Ryu[4], Leena Paul[1], Baranda S. Hansen[7], Khaled Khairy[1,6], Stephane Pelletier[8], Shondra M. Pruett-Miller[7,9], David R. Roalf[3], Raquel E. Gur[3], Beverly S. Emanuel[11], Donna M. McDonald-McGinn[11,12], Jesse N. Smith[13], Cai Li[13], Jason M. Christie[5,14], Paul A. Northcott[1], Stanislav S. Zakharenko[1,*]

**Affiliations**:

[1]Department of Developmental Neurobiology, St. Jude Children's Research Hospital, Memphis, TN 38105, USA

[2]Division of Neuroradiology, Department of Radiology, Hospital of the University of Pennsylvania, Philadelphia, PA 19104, USA

[3]Brain Behavior Laboratory, Neurodevelopment and Psychosis Section, Department of Psychiatry, University of Pennsylvania, Philadelphia, PA 19104, USA

[4]Center for In Vivo Imaging and Therapeutics, St. Jude Children's Research Hospital, Memphis, TN 38105, USA

[5]Max Planck Florida Institute for Neuroscience, Jupiter, FL 33458, USA

[6]Center for Bioimage Informatics, St. Jude Children's Research Hospital, Memphis, TN 38105, USA

[7]Center for Advanced Genome Engineering, St. Jude Children's Research Hospital, Memphis, TN 38105, USA

[8]Department of Medical and Molecular Genetics, Indiana University School of Medicine, Indianapolis, IN 46202, USA

[9]Department of Cell & Molecular Biology, St. Jude Children's Research Hospital, Memphis, TN 38105, USA

[11]Department of Pediatrics, Perelman School of Medicine, University of Pennsylvania, Philadelphia, PA; Division of Human Genetics, Children's Hospital of Philadelphia, Philadelphia, PA 19104, USA.

[12]Department of Molecular Medicine, Division of Human Biology and Medical Genetics, Sapienza University, Rome 00185, Italy

[13]Department of Biostatistics, St. Jude Children's Research Hospital, Memphis, TN 38105, USA

[14]Department of Physiology and Biophysics, University of Colorado Anschutz School of Medicine, Aurora, CO 80045, USA

***Correspondence:** stanislav.zakharenko@stjude.org

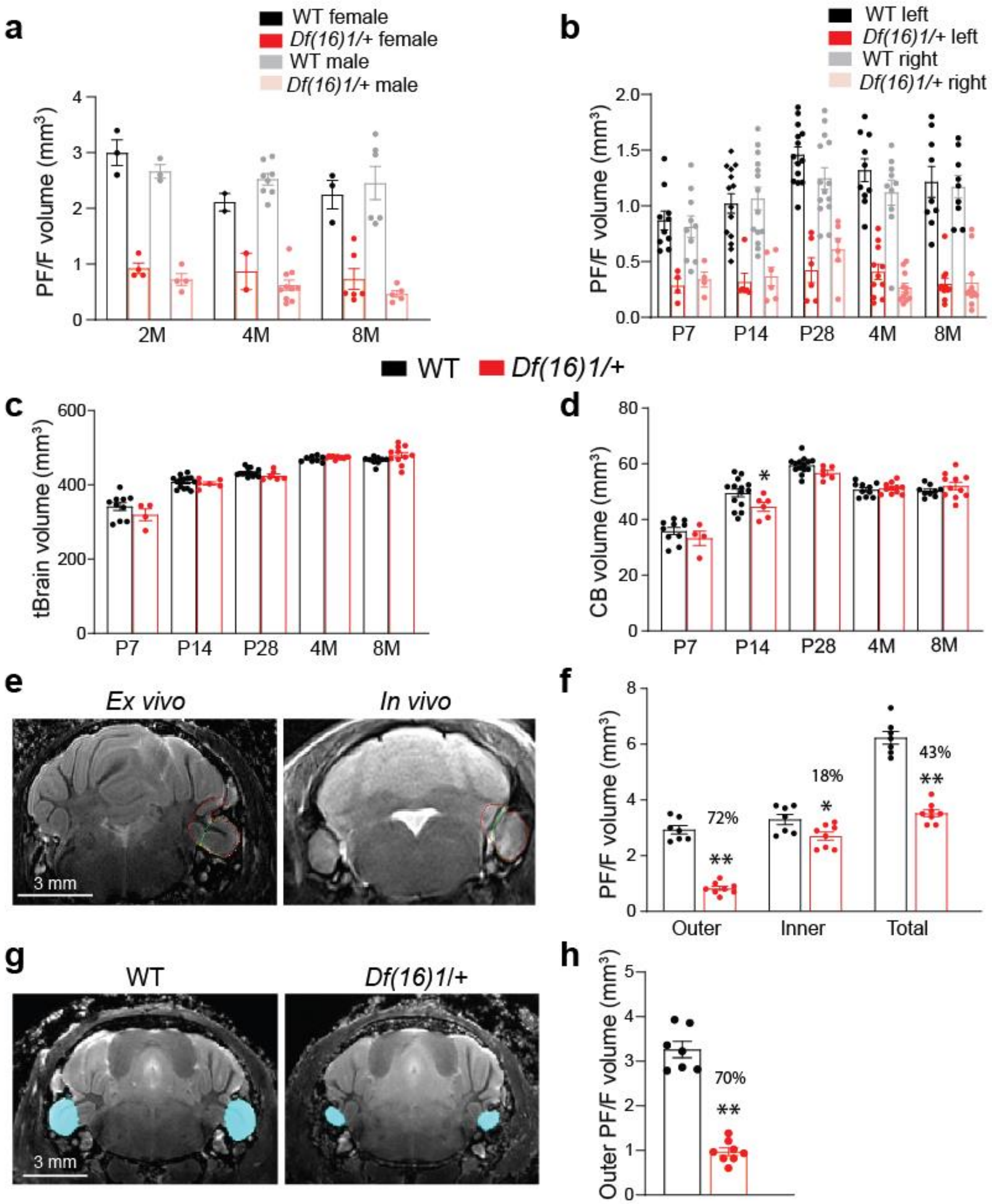


**Supplementary Figure 1. Characterization of the paraflocculus/flocculus dysplasia in *Df(16)1/+* mouse models of 22q11DS. a** Average PF/F volume in WT (black) and *Df(16)1/+* (red) male (gray, pink) and female (black, red) mice at different ages. At 2 months (M): female/male (WT 3/3 mice, *Df(16)1/+* 4/4 mice); 4M: WT 2/8, *Df(16)1/+* 2/10; 8M: WT 3/6, *Df(16)1/+* 6/5. Two-way ANOVA (Holm-Sidak's post-hoc); **a** $F_{6,\,44}$=0.79; *p*=0.58 (2M: *p*=0.5; 4M: *p*=0.41; 8M: *p*=0.45 from female vs male sex among *Df(16)1/+* mice). **b** Average left and right PF/F volumes in WT and *Df(16)1/+* mice at different ages. Postnatal day (P) 7: WT n=10, *Df(16)1/+* n=4; P14: WT n=14, *Df(16)1/+* n=6; P28: WT n=14, *Df(16)1/+* n=6; 4M: WT n=10, *Df(16)1/+* n=11; 8M: WT n=9, *Df(16)1/+* n=11. Two-way ANOVA (Holm-Sidak's post-hoc); **b** $F_{12,}$

$_{170}$=1.68; $p$=0.08 (P7: $p$=0.91; P14: $p$=0.92; P28: $p$=0.32; 4M: $p$=0.3; 8M: $p$=0.95 from left (black, red) vs right (gray, pink) PF/F volume in *Df(16)1/+* mice). **c**, **d** Average total brain volume in WT and *Df(16)1/+* mice at different ages. P7: WT n=10, *Df(16)1/+* n=4; P14: WT n=14, *Df(16)1/+* n=6; P28: WT n=14, *Df(16)1/+* n=6; 4M: WT n=8, *Df(16)1/+* n=11; 8M: WT n=9, *Df(16)1/+* n=11. Two-way ANOVA (Holm-Sidak's post-hoc); **c** $F_{1, 83}$=0.64; $p$=0.43 (P7: $p$=0.19; P14: $p$=0.84; P28: $p$=0.69; 4M: $p$=0.84; 8M: $p$=0.28). **d** Average volumes of total cerebellum (CB) in WT and *Df(16)1/+* mice at different ages. P7: WT n=10, *Df(16)1/+* n=4; P14: WT n=14, *Df(16)1/+* n=6; P28: WT n=14, *Df(16)1/+* n=6; 4M: WT n=10, *Df(16)1/+* n=11; 8M: WT n=9, *Df(16)1/+* n=11. Two-way ANOVA (Holm-Sidak's post-hoc); **d** $F_{1, 85}$=4.07; $p$=0.047 (P7: $p$=0.54; P14: *$p$=0.03; P28: $p$=0.41; 4M: $p$=0.78; 8M: $p$=0.54). **e** Representative MRIs of *ex vivo* and *in vivo* cerebella containing the PF/Fs with the outer (red lines) and inner parts manually outlined (green lines divide inner and outer parts of the PF/F). **f** Average PF/F volumes based on outer, inner, and total manual measurements in WT (n=10) and *Df(16)1/+* (n=8) mice. Two-tailed Student's *t*-test: $t_{13}$=13.01, **$p$ <0.0001, a 72% decrease in the outer PF/F volume in *Df(16)1/+* mice; $t_{13}$=10.61, *$p$=0.02, an 18% decrease in the inner PF/F volume in *Df(16)1/+* mice; $t_{13}$=2.57, **$p$ <0.0001, a 43% decrease in the total PF/F volume in *Df(16)1/* mice compared to WT littermates. **g** Representative MRIs of WT and *Df(16)1/+* cerebella *in vivo*; the outer PF/Fs are automatically outlined (turquoise). **h** Average volume of the outer PF/F based on automatic measurement in WT (n=7) and *Df(16)1/+* (n=8) mice. Two-tailed Student's *t*-test; $t_{13}$=11.87, **$p$ <0.0001, a 70% decrease in *Df(16)1/+* mice compared to WT littermates. Data are presented as the mean ± SEM. Source data are provided as a Source Data file.

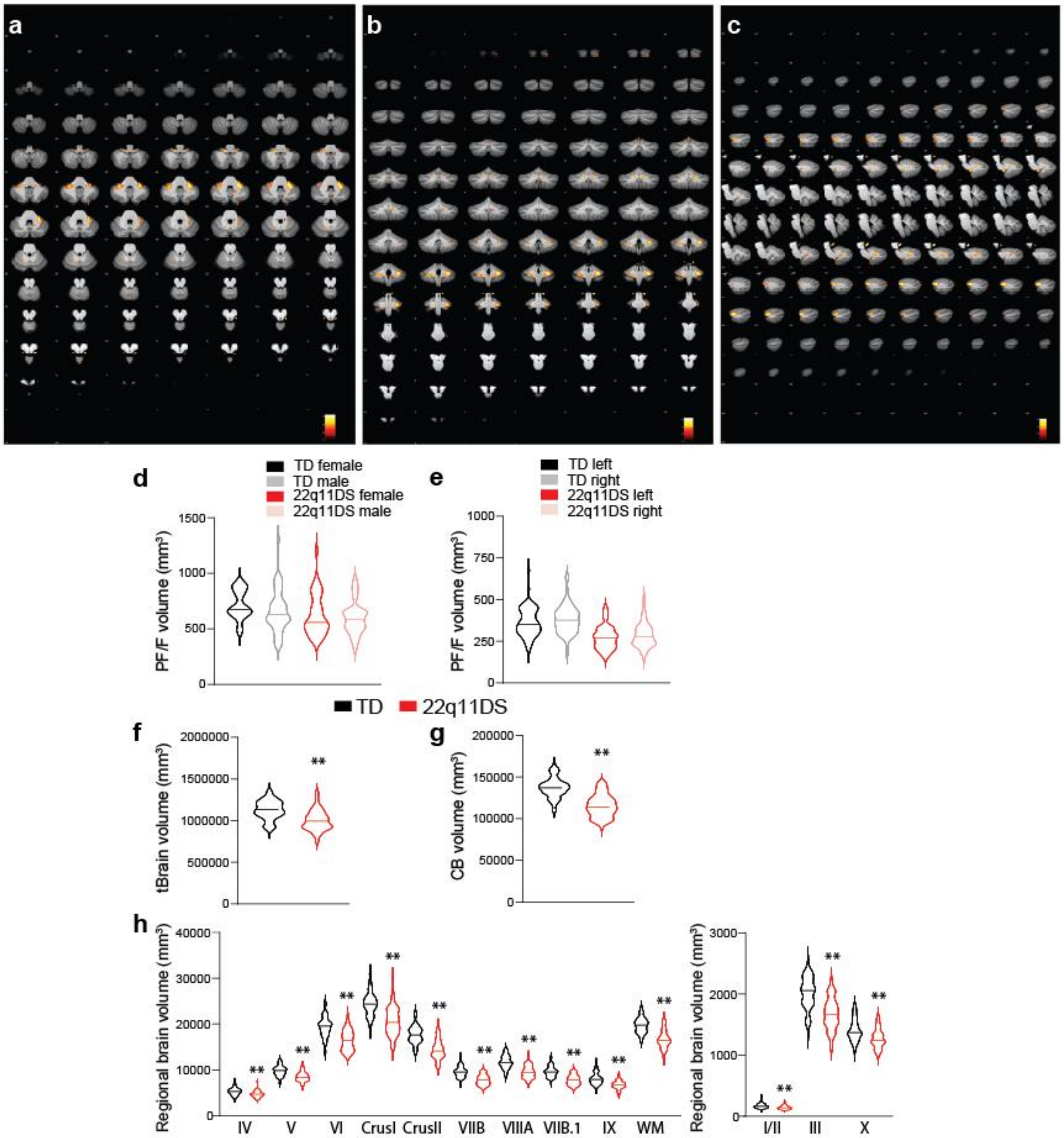


**Supplementary Figure 2. Characterization of the cerebellum in individuals with 22q11DS. a-c** Probability maps from cerebellar voxel-based morphometry (VBM) on axial (**a**), coronal (**b**), and sagittal (**c**) slices were overlaid on the entire cerebellum. F-scores depicted by false colors indicate morphologic differences in subjects with 22q11DS (red) compared to TD controls (black). **d** PF/F volumes in human males (gray, pink, n=38) and females (black, red, n=42) with 22q11DS and in TD males (n=42) and females (n=26). Two-tailed Student's *t*-test. TD: $t_{66}$=1.32, $p$=0.19; 22q11DS: $t_{78}$=0.76, $p$=0.44. **e** Left (black, red) and right (gray, pink, PF/F volumes in TD controls (n=68) and individuals with 22q11DS (n=80). Two-tailed Student's *t*-test. TD: $t_{134}$=1.12, $p$=0.26; 22q11DS: $t_{158}$=1.22, $p$=0.22. **f**, **g** Total-brain (tBrain; **f**) and cerebellum (CB; **g**) volumes in TD controls (n=68) and individuals with 22q11DS (n=79). Two-tailed Student's *t*-test. tBrain: $t_{145}$=6.26, **$p$ <0.0001; CB: $t_{145}$=10.26, **$p$ <0.0001. **h** Regional cerebellar volumes were measured by MAGeT in TD controls (n=68) and individuals with 22q11DS (n=80). Two-way ANOVA (Holm-Sidak's post-hoc); $F_{19,\ 1372}$=1161; **$p$=0.0001. Data are presented as violin plots with median values. Source data are provided as a Source Data file.

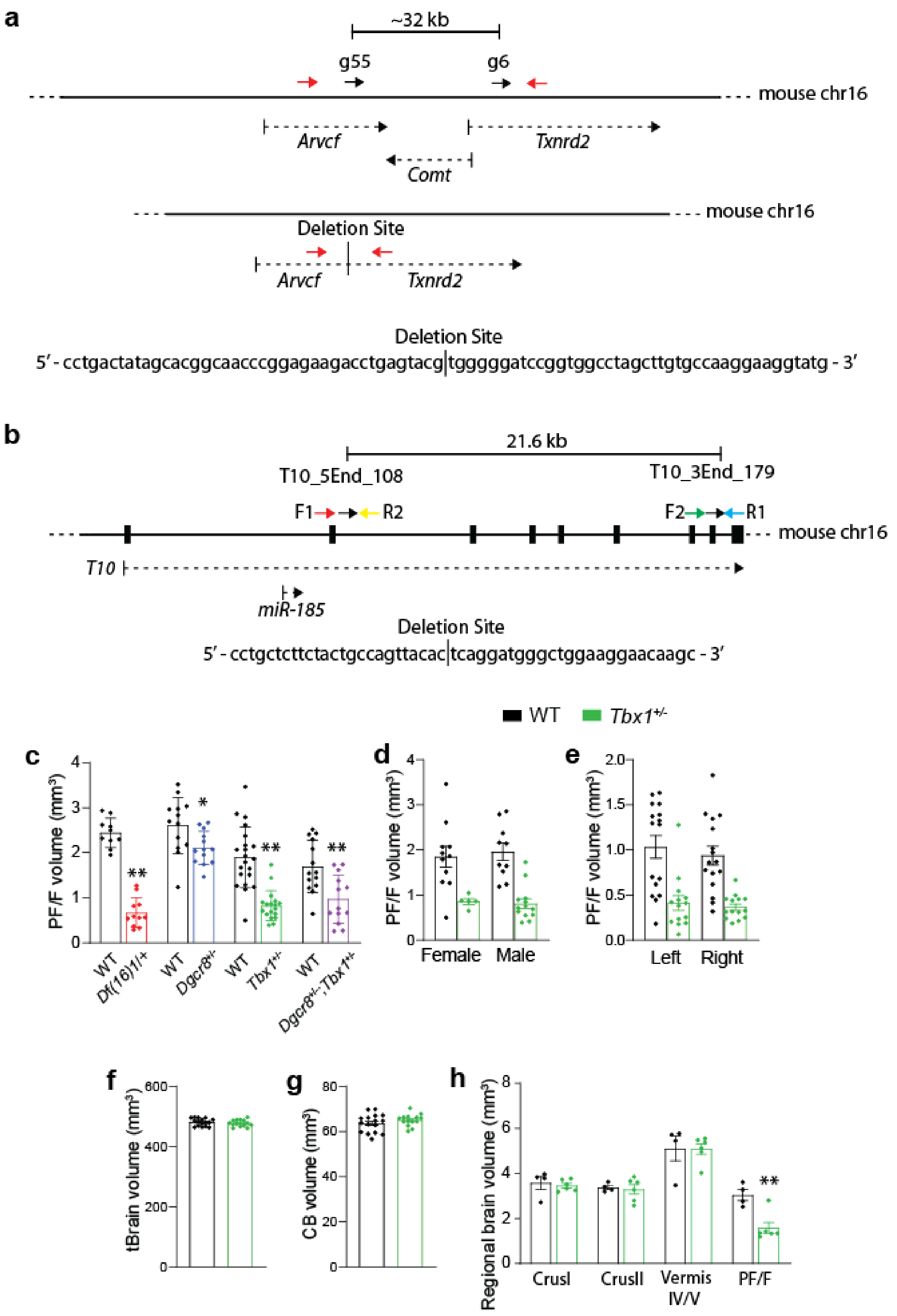
a
~32 kb
g55
g6
mouse chr16
Arvcf
Txnrd2
Comt
mouse chr16
Deletion Site
Arvcf
Txnrd2
Deletion Site
5′ - cctgactatagcacggcaacccggagaagacctgagtacg|tggggatccggtggcctagcttgtgccaaggaaggtatg - 3′
b
21.6 kb
T10_5End_108
T10_3End_179
F1
R2
F2
R1
mouse chr16
T10
miR-185
Deletion Site
5′ - cctgctcttctactgccagttacac|tcaggatgggctggaaggaacaagc - 3′
WT
Tbx1+/-
c
PF/F volume (mm3)
WT
Df(16)1/+
WT
Dgcr8+/-
WT
Tbx1+/-
WT
Dgcr8+/-;Tbx1+/-
d
PF/F volume (mm3)
Female
Male
e
PF/F volume (mm3)
Left
Right
f
tBrain volume (mm3)
g
CB volume (mm3)
h
Regional brain volume (mm3)
CrusI
CrusII
Vermis IV/V
PF/F

**Supplementary Figure 3. Screening of genes responsible for the paraflocculus/flocculus dysplasia in 22q11DS mice and characterization of the cerebellum of *Tbx1*$^{+/–}$ mice. a** Strategy for generating the *Arvcf-Comt-Txnrd2*–deletion mouse. (Top) CRISPR-Cas9 and direct zygote injection were used to engineer a mouse model with an ~32-kb deletion in chromosome 16, between the *Arvcf* and *Txnrd2* genes. The cut site for g55 sgRNA is within coding exon 3 of *Arvcf*, and the cut site for g6 sgRNA is within coding exon 2 of *Txnrd2*. The resulting deletion creates a fusion protein with a premature stop codon to knock out expression of *Arvcf, Comt* (completely deleted), and *Txnrd2*. Genotyping primers for deletion-positive pups are shown in red. (Bottom) Sequence of the resulting deletion mouse with the deletion site marked. **b** Strategy for generating the *T10* (*Tango 2*) knockout mouse model. (Top) *T10*-knockout mice were engineered by creating a ~21.6-kb deletion encompassing intron 2 to intron 8 of *T10* by using CRISPR-Cas9 technology delivered in pronuclear-stage zygotes. The resulting deletion removes >80% of *T10*-coding sequence but leaves *miR185*-encoding sequences intact. The allele was engineered such that splicing from exon 2 into exon 9 is in frame, avoiding degradation of the transcript via nonsense-mediated mRNA decay and preserving *miR185* expression. Colored arrows represent PCR-genotyping primers. Black arrows indicate the location of the cut sites by the two sgRNAs. Black dashed arrows indicate genes. Black boxes represent *T10* exons. (Bottom) Sequence of the resulting deletion mouse with the deletion site marked. **c** PF/F volumes in *Df(16)1/+* (red, n=11), *Dgcr8*$^{+/–}$ (blue, n=13), *Tbx1*$^{+/–}$ (green, n=18), and *Dgcr8*$^{+/–}$;*Tbx1*$^{+/–}$ (purple, n=13) mice and their respective WT littermates (n=10, 13, 21, 14, respectively). Two-tailed Student's *t*-test. *Df(16)1/+*: $t_{19}$=12.39, $**p$ <0.0001; *Dgcr8*$^{+/–}$: $t_{24}$=2.49, $*p$=0.02; *Tbx1*$^{+/–}$: $t_{37}$=6.15, $**p$ <0.0001; *Dgcr8*$^{+/–}$;*Tbx1*$^{+/–}$: $t_{24}$=6.29, $**p$ <0.0001. **d** PF/F volumes in 2- to 4-month-old female and male WT and *Tbx1*$^{+/–}$ mice. Female/male: WT 11/10 mice, *Tbx1*$^{+/–}$ 5/13 mice. Two-tailed Student's *t*-test; WT: $t_{19}$=1.43, $p$=0.36; *Tbx1*$^{+/–}$: $t_{16}$=0.27, $p$=0.79. **e** Volumes of the left and right PF/Fs in 2- to 4-month-old WT (n=16) and *Tbx1*$^{+/–}$ (n=14) mice. Two-tailed Student's *t*-test. WT: $t_{30}$=0.59, $p$=0.56; *Tbx1*$^{+/–}$: $t_{26}$=0.51, $p$=0.61. **f** Total-brain (tBrain) volume in 2- to 4-month-old WT (n=16) and *Tbx1*$^{+/–}$ (n=14) mice. Two-tailed Student's *t*-test; $t_{28}$=0.91, $p$=0.37. **g** Cerebellar (CB) volume in 2- to 4-month-old WT (n=16) and *Tbx1*$^{+/–}$ (n=14) mice. Two-tailed Student's *t*-test: $t_{28}$=1.1, $p$=0.28. **h** Regional cerebellar volumes in WT (n=4) and *Tbx1*$^{+/–}$ (n=6) mice. Two-tailed Student's *t*-test. Crus I: $t_8$=0.44, $p$=0.67; Crus II: $t_8$=0.3, $p$=0.77; Vermis IV/V: $t_8$=0.04, $p$=0.97; PF/F: $t_8$=4.02, $**p$ <0.01. Data are presented as the mean ± SEM. Source data are provided as a Source Data file.

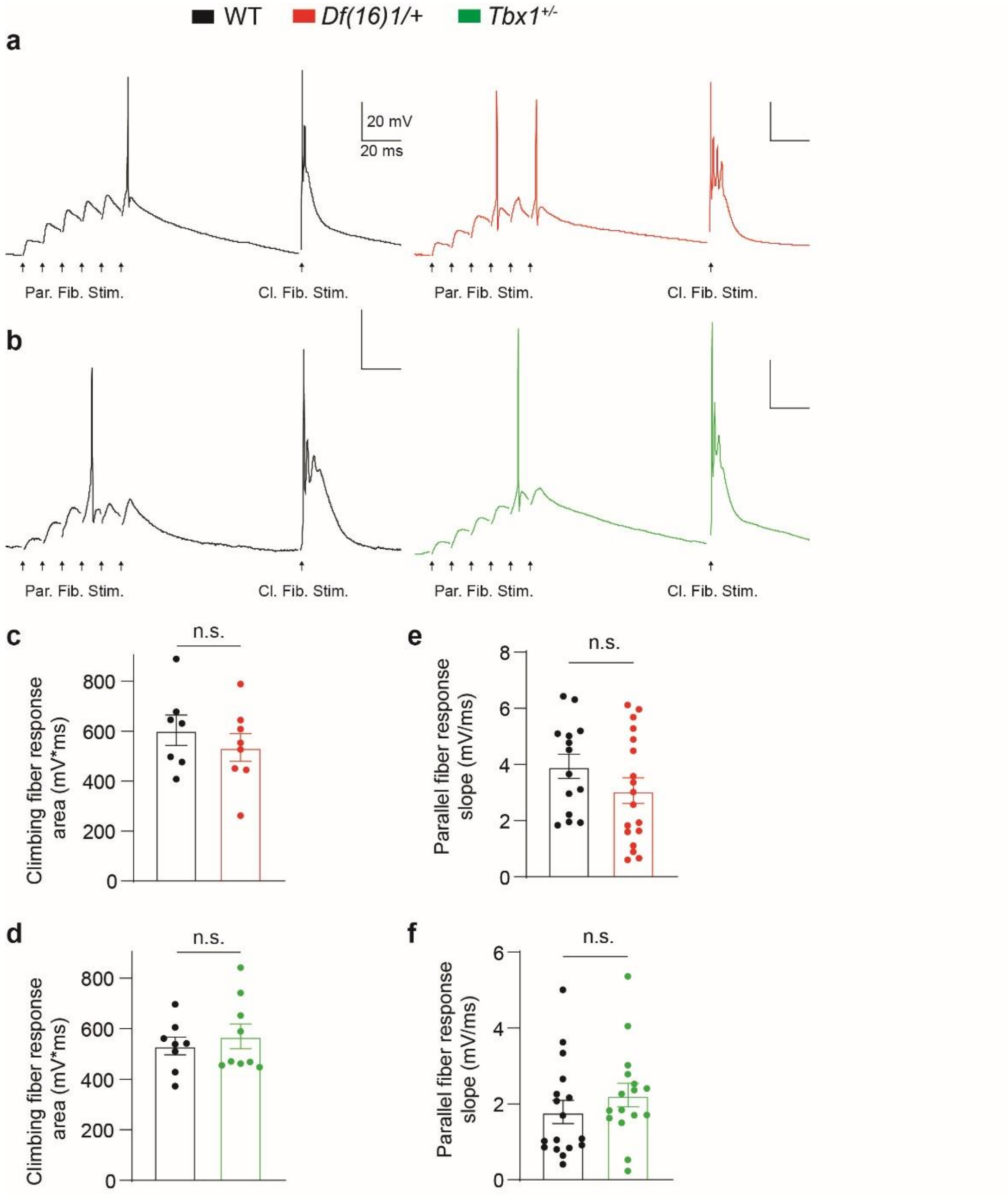


**Supplementary Figure 4. Normal climbing fiber and parallel fiber responses in *Df(16)1*/+ and *Tbx1*$^{+/-}$ mice. a, b** Representative traces of voltage responses recorded during LTD induction in WT (black) and *Df(16)1*/+ (red) (**a**) and WT and *Tbx1*$^{+/-}$ (green) (**b**) PCs. Parallel fiber tetanic stimulation results in EPSPs, occasionally culminating in simple spikes. Climbing fiber stimulation results in a complex spike. **c** Average climbing fiber response area during LTD induction in WT (n=7 cells from 4 mice) and *Df(16)1*/+ (n=8 cells, 5 mice) PCs. Two-tailed Student's *t*-test (Welch's correction): $t_{12.63}$=0.84, $p$=0.418. **d** Average climbing fiber response area during LTD induction in WT (n=8 cells, 5 mice) and *Tbx1*$^{+/-}$ (n=9 cells, 6 mice) PCs. Two-tailed Student's *t*-test (Welch's correction): $t_{14.17}$=0.63, $p$=0.537. **e** Average baseline parallel fiber response slope in WT (n=14 cells, 9 mice) and *Df(16)1*/+ (n=18 cells, 9 mice) PCs. Two-tailed Student's *t*-test (Welch's correction): $t_{29.78}$=1.37, $p$=0.18. **f** Average baseline parallel fiber response slope in WT (n=17 cells, 11 mice) and *Tbx1*$^{+/-}$ (n=16 cells, 9 mice) PCs. Two-tailed Student's *t*-test (Welch's correction): $t_{30.96}$=1.02, $p$=0.316. Data are presented as the mean ± SEM. Abbreviation: n.s., not significant. Source data are provided as a Source Data file.

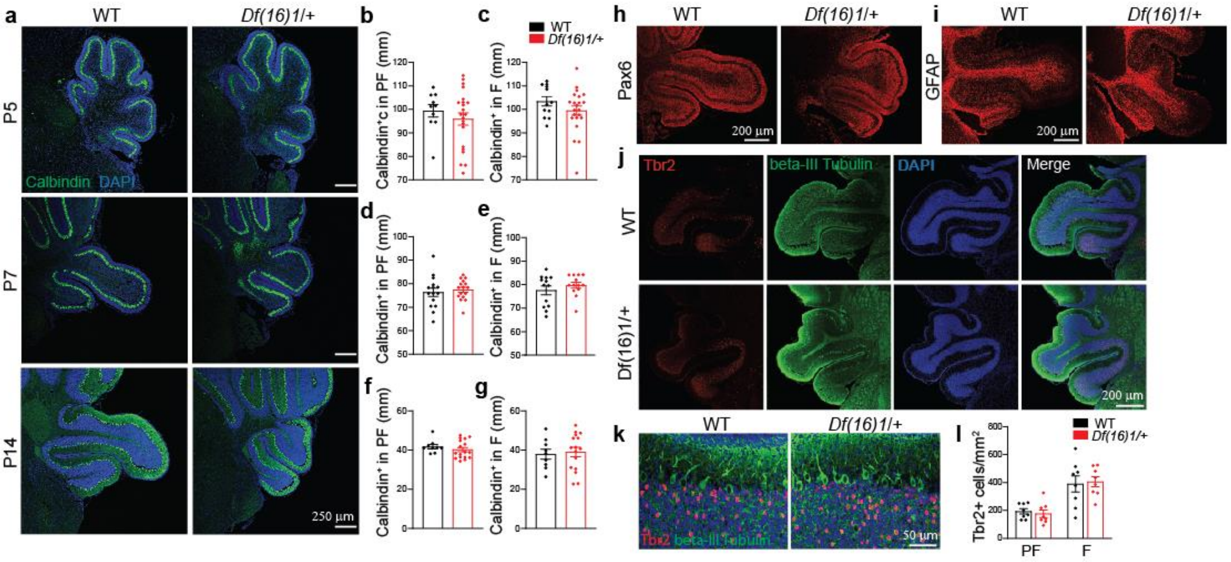


**Supplementary Figure 5. Cellular composition is not altered in the paraflocculus/flocculus of 22q11DS mice. a-g** Representative confocal images of calbindin (Purkinje cell marker, green) and DAPI (nuclear marker, blue) staining in the coronal cerebellar sections from P5–P14 WT (black) and *Df(16)1/+* (red) mice. Mean calbindin$^+$ cell density in the PF (**b**, **d**, **f**) and F (**c**, **e**, **g**) at P5 (**b**, **c**), P7 (**d**, **e**), and P14 (**f**, **g**) in WT (**b** 10 sections, 2 mice; **c** 12 sections, 2 mice; **d** 15 sections, 5 mice, **e** 13 sections, 5 mice; **f** 9 sections, 2 mice; **g** 9 sections, 2 mice) and *Df(16)1/+* (**b** 22 sections, 5 mice; **c** 22 sections, 5 mice; **d** 16 sections, 5 mice, **e** 13 sections, 5 mice; **f** 17 sections, 4 mice; **g** 16 sections, 4 mice) animals. Two-tailed Student's *t*-test. P5: **b** $t_{30}$=0.82, p=0.42, **c** $t_{32}$=1.29, p=0.21, **d** P7:$t_{29}$=0.53, **e** p=0.6, $t_{24}$=0.1, p=0.33, **f** P14: $t_{24}$=0.87, p=0.39, **g** $t_{23}$=0.3, p=0.77. **h**, **i** Representative confocal images of Pax6 (**h**, granular progenitor marker, red) and GFAP (**i**, astrocyte marker, red) staining in the coronal cerebellar sections in P14 WT and *Df(16)1/+* mice. **j**, **k** Representative low- (**j**) and high-magnification (**k**) confocal images of Tbr2 (unipolar brush cell marker, red), beta-III-tubulin (neuronal marker, green), and DAPI staining in coronal cerebellar sections of P14 WT and *Df(16)1/+* mice. Note the high density of Tbr2$^+$ cells in the PF/F in **k**. **l** Average numbers of Tbr2$^+$ cells in the PF/F of WT (8 brain sections, 5 mice) and *Df(16)1/+* (8 brain sections, 5 mice) animals. Two-tailed Student's *t*-test. PF: $t_{14}$=0.51, *p*=0.62, F: $t_{14}$=0.23, *p*=0.82. Data are presented as the mean ± SEM. Source data are provided as a Source Data file.

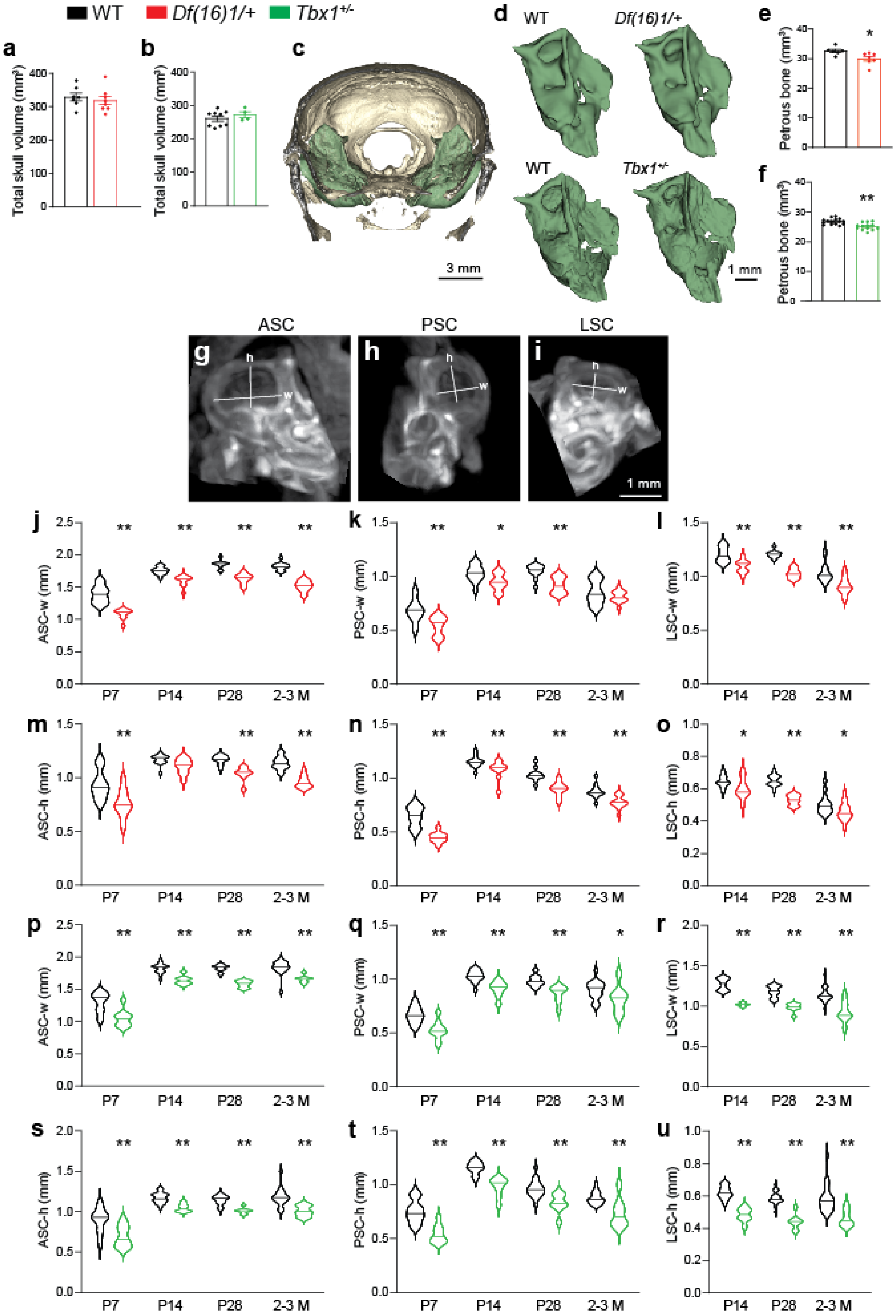

WT
Df(16)1/+
Tbx1+/-
a
b
c
d
e
f
Total skull volume (mm³)
Petrous bone (mm³)
3 mm
1 mm
ASC
PSC
LSC
g
h
i
j
k
l
m
n
o
p
q
r
s
t
u
ASC-w (mm)
PSC-w (mm)
LSC-w (mm)
ASC-h (mm)
PSC-h (mm)
LSC-h (mm)
P7
P14
P28
2-3 M

**Supplementary Figure 6. Petrous temporal bone and semicircular canals are reduced in *Df(16)1/+* and *Tbx1*$^{+/–}$ mice. a**, **b** Total-skull volume in the *Df(16)1/+* mice (**a** n=8, red), *Tbx1*$^{+/–}$ mice (**b** n=4, green), and their respective WT controls (**a** n=7, **b** n=9, black). Two-tailed Student's *t*-test; **a** $t_{13}$=0.64, **b** $p$=0.53; $t_{11}$=0.79, $p$=0.45. **c**, **d** The 3D rendering of the PTB (green) in the skull (golden) (**c**) and those of the PTBs in *Df(16)1/+* mice and *Tbx1*$^{+/–}$ mice compared to their matched WT mice (**d**). **e**, **f** Average PTB volumes in *Df(16)1/+* mice (n=7) and *Tbx1*$^{+/–}$ mice (n=13) and their matched WT controls (n=6, 15). Two-tailed Student's *t*-test. **e** $t_{11}$=2.61, *$p$=0.02; **f** $t_{26}$=4.06, **$p$=0.0004. **g-i** Representative CT images of the ASC (**g**), PSC (**h**), and LSC (**i**), with corresponding width (w) and height (h) measures labeled. **j-u** Width and height of each canal in *Df(16)1/+* mice (**j-o** 7-16 mice), *Tbx1*$^{+/–}$ mice (**p-u** 7-10 mice), and their respective WT littermates (**j-o** 12-14 mice, **p-u** 17-18 mice) at different ages. Two-way ANOVA (Holm-Sidak's post-hoc); **j** $F_{1, 90}$=199.7; **$p$ <0.0001 (P7: **$p$ <0.0001; P14: **$p$ <0.0001; P28: **$p$ <0.0001; 2-3 M: **$p$ <0.0001); **k** $F_{1, 89}$=31.89; **$p$ <0.0001 (P7: **$p$=0.0006; P14: *$p$=0.037; P28: **$p$=0.003; 2-3 M: $p$=0.2); **l** $F_{1, 66}$=65.1; **$p$ <0.0001 (P14: **$p$=0.0004; P28: **$p$ <0.0001; 2-3 M: **$p$ <0.0001); **m** $F_{1, 90}$=64.81; **$p$<0.0001 (P7: **$p$ <0.0001; P14: $p$=0.066; P28: **$p$=0.002; 2-3 M: **$p$ <0.0001); **n** $F_{1, 89}$=81.08; **$p$ <0.0001 (P7: **$p$ <0.0001; P14: **$p$=0.007; P28: **$p$=0.0001; 2-3 M: **$p$=0.0004); **o** $F_{(1, 66)}$ =32.83; **$p$ <0.0001 (P14: *$p$=0.04; P28: **$p$ <0.0001; 2-3 M: *$p$=0.033); **p** $F_{1, 96}$=109.4; **$p$ <0.0001 (P7: **$p$ <0.0001; P14: **$p$< 0.0001; P28: **$p$ <0.0001; 2-3 M: **$p$ <0.0001); **q** $F_{1, 96}$=54.2; **$p$ <0.0001 (P7: **$p$ <0.0001; P14: **$p$=0.0008; P28: **$p$=0.0007; 2-3 M: *$p$=0.01); **r** $F_{1, 72}$=126.1; **$p$ <0.0001 (P14: **$p$ <0.0001; P28: **$p$ <0.0001; 2-3 M: **$p$ <0.0001); **s** $F_{1, 96}$=58.87; **$p$ <0.0001 (P7: **$p$ <0.0001; P14: **$p$=0.004; P28: **$p$=0.003; 2-3 M: **$p$=0.0003); **t** $F_{1, 96}$=87.04; **$p$ <0.0001 (P7: **$p$ <0.0001; P14: **$p$ <0.0001; P28: **$p$=0.004; 2-3 M: **$p$=0.0004); **u** $F_{1, 71}$=94.02; $p$ <0.0001 (P14: **$p$ <0.0001; P28: **$p$ <0.0001; 2-3 M: **$p$ <0.0001). Data are presented as the mean ± SEM (**a**, **b**, **e**, **f**) or as violin plots with median values (**j-u**). Source data are provided as a Source Data file.

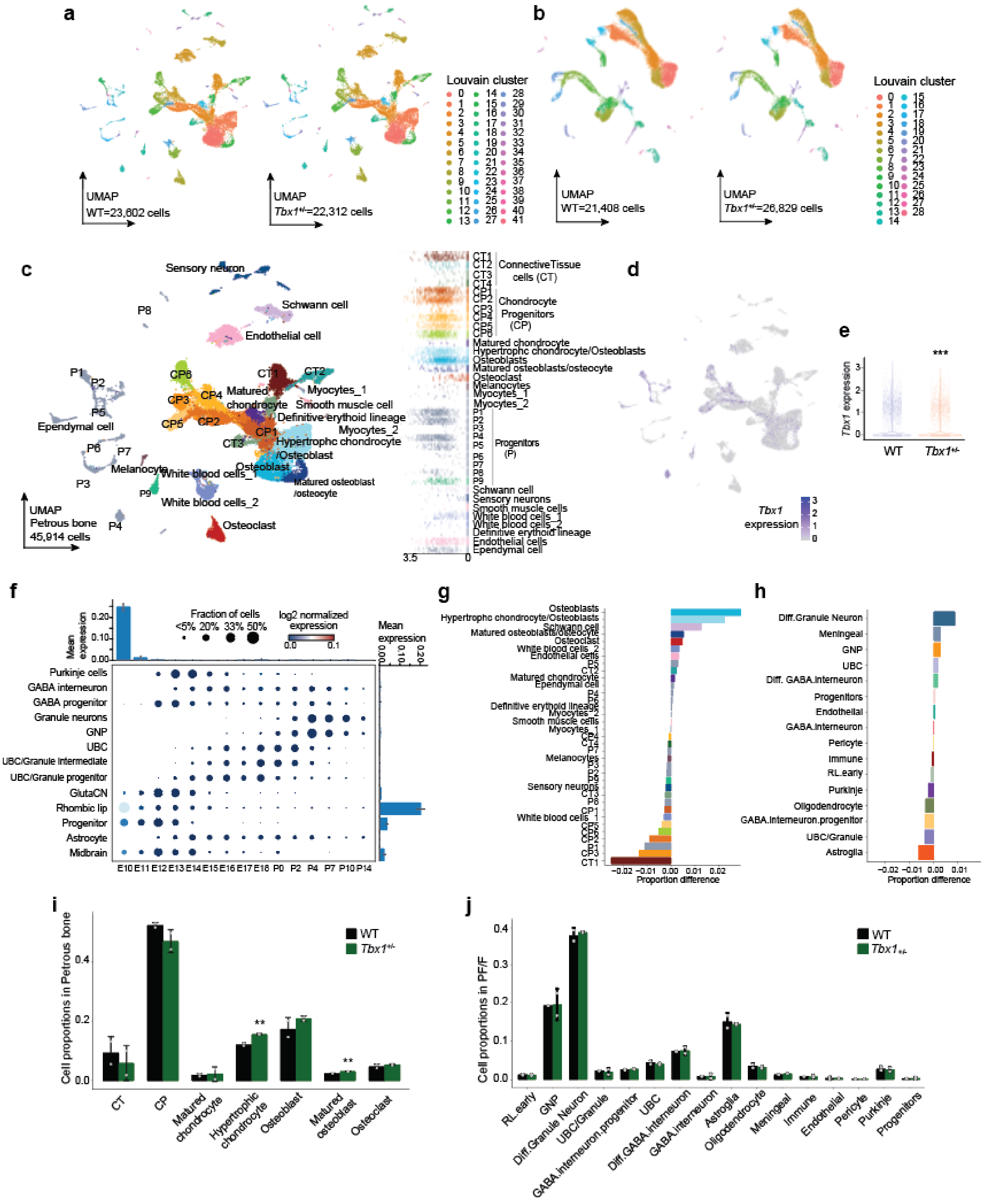


**Supplementary Figure 7. snRNA-seq profiling reveals a selective effect of *Tbx1* haploinsufficiency on cell populations in the petrous temporal bone but not in the paraflocculus/flocculus. a** UMAP visualizations of cell populations in the PTB from WT (left, 23,602 cells) and *Tbx1*$^{+/-}$ (right, 22,312 cells) samples. Each plot displays Louvain clusters that are annotated and color-coded to represent distinct cell populations in the PTB samples. **b** UMAP visualizations of cell populations in the PF/F from WT (left,

21,408 cells) and *Tbx1*$^{+/–}$ (right, 26,829 cells) mice. Louvain clusters are annotated and color-coded to represent distinct cell populations in the PF/F samples. **c** Combined and annotated UMAP representation (left) of total cell types (45,914 cells) isolated from the PTB samples. Specific cell types, including multiple progenitor subpopulations (P), connective tissue (CT) subpopulations, chondrocyte progenitors (CP), different state of CT and osteoblasts, and various immune cells are labeled and color-coded for identification. Dot plot (right) shows individual cells color-coded by cell type, with the x-axis denoting the level of *Tbx1* expression, and the y-axis noting the cell types. **d** UMAP visualization of the distribution and expression levels of *Tbx1* across all cell types in the PTB. **e** A comparison of *Tbx1* expression levels in the chondrocyte–osteoblast populations between WT (blue) and *Tbx1*$^{+/–}$ (red) mice. Wilcoxon test: $Log_2$ FC =–0.05, ***FDR =5.08e-4. **f** *Tbx1* expression in developing WT murine cerebellar cell types. Bubble chart data represent the fraction and mean $Log_2$-normalized levels of *Tbx1* in cells with non-zero *Tbx1* expression, across various cerebellar cell types and developmental stages. Bubble size corresponds to the percentage of cells expressing *Tbx1* within each cell type and at each stage. Mean expression was calculated only from cells with detectable *Tbx1* expression. **g** Proportional differences in all cell types in the PTB between WT and *Tbx1*$^{+/–}$ mice. The x-axis represents the difference in cell proportions, calculated by cell proportion of *Tbx1*$^{+/–}$ minus that of WT. Positive values (to the right) indicate a higher cell proportion in the *Tbx1*$^{+/–}$ sample, and negative values (to the left) suggest a lower proportion in the *Tbx1*$^{+/–}$ sample compared to the WT. **h** Proportional differences in all cell types in the PF/F between WT and *Tbx1*$^{+/–}$ mice. The cell-type proportions showed minimal variation, with clear lineage or differentiation patterns either lacking or not detected in the PF/F. **i** Comparisons of chondro-osteogenic cell-type proportions isolated from the PTB of WT mice and *Tbx1*$^{+/–}$ mice. DCATS (beta-binomial regression-based test); **FDR <0.01. **j** Comparisons of individual cerebellar cell–type compositions isolated from the PF/F of WT (black) and *Tbx1*$^{+/–}$ (green) mice. Standard deviation error bars represent variability between two experimental batches in **i**, **j**. The statistical significance for each cell type was determined using DCATS, with cell types exhibiting an FDR <0.1 were considered significant. Abbreviations: FC, fold change; FDR, false discovery rate. Source data are provided as a Source Data file.

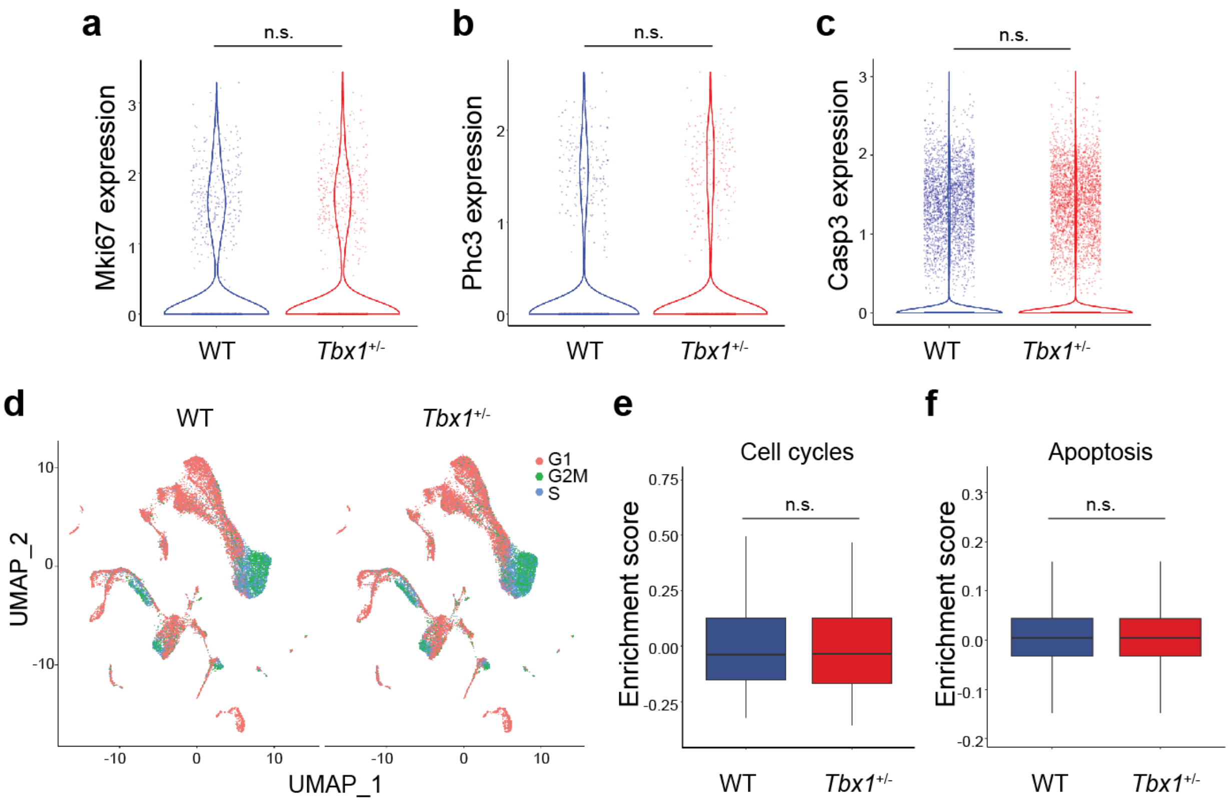


**Supplementary Figure 8. snRNA-seq profiling shows no difference in the expression of gene markers related to cell proliferation, mitosis, cell cycle, or apoptosis in the PF/F between WT mice and *Tbx1*$^{+/–}$ mice.** Cell populations in the P5.5 PF/F from WT (blue) and *Tbx1*$^{+/–}$ (red) mice are compared for the expression of the cell proliferation marker *Mki67* (**a**), mitotic marker *Phc3* (**b**), and apoptosis marker *Casp3* (**c**), showing no significant difference between the two groups. **a** *p*=0.39, adjusted *p*-value=1, $log_2$FC=-0.05; *Mki67* expression >0: 41.8% in WT mice (GNP 4,129 cells) vs. 41.9% in *Tbx1*$^{+/–}$ mice (GNP 5,275 cells). **b** *p*=0.11, adjusted *p*-value=1, $log_2$FC=-0.03; *Phc3* expression >0: 19.2% in WT mice (GNP 4,129 cells) vs. 17.7% in *Tbx1*$^{+/–}$ mice (GNP 5,275 cells). **c** *p*=0.003, adjusted *p*-value=1, $log_2$FC=-0.02; *Casp3* expression >0: 12.2 % in WT mice (21,408 cells) vs. 11.3% in *Tbx1*$^{+/–}$ mice (26,829 cells). **d** UMAP of PF/F in WT and *Tbx1*$^{+/–}$ mice, colored by predicted cell cycle phase from Seurat, shows no significant difference in the proportion of cycling cells between the two groups. **e** Cell cycling gene set enrichment score (94 cycling-related genes in the Seurat package) in WT (21,408 cells) and *Tbx1*$^{+/–}$ mice (26,829 cells): *p*=0.01, with a percentage of enrichment score–positive cells of 27.3% in WT mice vs. 27.7% in *Tbx1*$^{+/–}$ mice. **f** Apoptosis gene set enrichment score (85 apoptosis-related genes in KEGG) in WT (21,408 cells) and *Tbx1*$^{+/–}$ mice (26,829 cells): *p*=0.05, with a percentage of enrichment score–positive cells of 52.5% in WT mice vs. 52.1% in *Tbx1*$^{+/–}$ mice. Significance cutoff: adjusted *p*-value <0.05, FC >1.5, difference in the proportion of positive cells >10%. n.s., not significant. Source data are provided as a Source Data file.

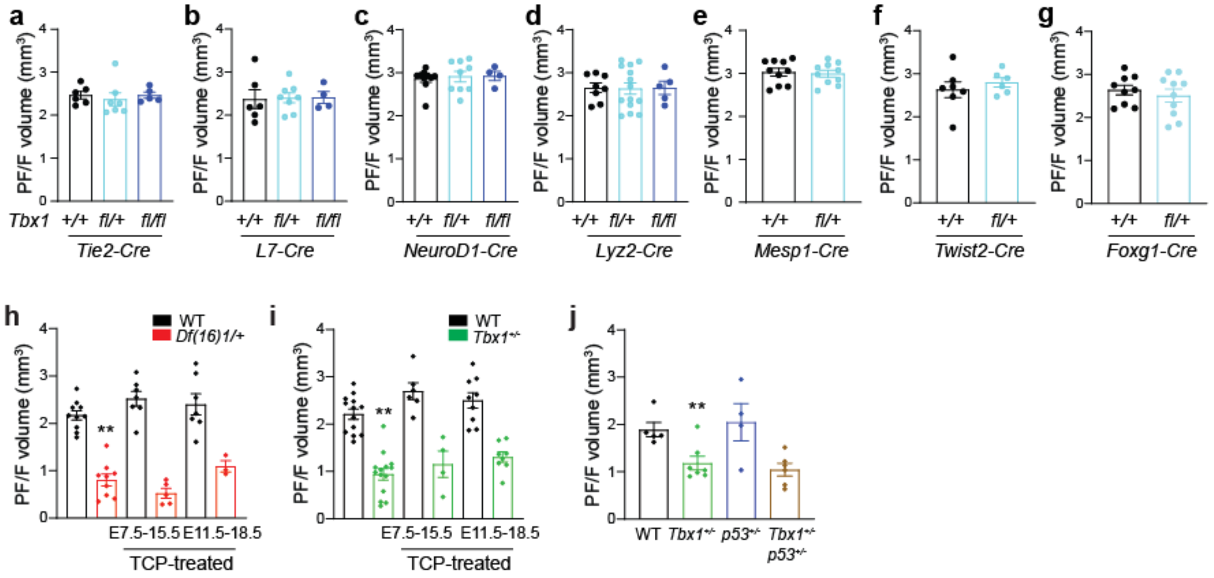


**Supplementary Figure 9. Approaches to mimic or rescue paraflocculus/flocculus dysplasia. a-g** Total PF/F volumes in mice with conditional *Tbx1* deletion (WT, black; hemizygous deletion, turquoise; homozygous deletion, blue) in endothelial cells (*Tie2$^{Cre}$* mice) (**a** WT n=6, heterozygous n=7, homozygous n=5), Purkinje cells (*L7$^{Cre}$* mice) (**b** WT n=6, heterozygous n=8, homozygous n=4), granule cells using *NeuroD1$^{Cre}$* mice (**c** WT n=10, heterozygous n=9, homozygous n=4), osteoclasts (*Lyz2$^{Cre}$* mice) (**d** WT n=8, heterozygous n=14, homozygous n=5), mesodermal cells (*Mesp1$^{Cre}$* mice) (**e** WT n=10, heterozygous n=10), osteoprogenitors (*Twist2$^{Cre}$* mice) (**f** WT n=7, heterozygous n=6), and otic vesicle progenitors (*Foxg1$^{Cre}$* mice) (**g** WT n=9, heterozygous n=10). One-way ANOVA. **a** $F_{2, 15}$=0.24; *p*=0.79; **b** $F_{2, 15}$=0.02; *p*=0.98; **c** $F_{2, 20}$=0.12; *p*=0.89; **d** $F_{2, 24}$=0.01; *p*=0.99. Two-tailed Student's *t*-test. **e** $t_{18}$=0.29, p=0.77; **f** $t_{11}$=0.76, p=0.46; **g** $t_{17}$=0.66, p=0.52. **h, i** PF/F volumes in nontreated vs tranylcypromine (TCP)-treated *Df(16)1/+* mice (red, **h** n=9, 5, 3), *Tbx1$^{+/–}$* mice (green, **i** n=14, 4, 8), and their respective WT littermates (black, **h** n=10, 7, 7; **i** n=13, 6, 9). Two-way ANOVA (Holm-Sidak's post-hoc). **h** $F_{5, 26}$=31.75; ***p* <0.0001; **i** $F_{5, 35}$=22.98; ***p* <0.0001. **j** PF/F volumes in *Tbx1$^{+/–}$* mice (n=7), *Tbx1$^{+/–}$;p53$^{+/–}$* mice (brown, n=6), and their WT (n=5) and *p53$^{+/–}$* (blue, n=4) littermates. One-way ANOVA (Sidak's post-hoc). $F_{3, 18}$=6; ***p* <0.004. Data are presented as the mean ± SEM. Source data are provided as a Source Data file.

**Supplementary Table 1.** Summary Statistics from SUIT-VBM.

| *set-level* | | *cluster-level* | | | | *peak-level* | | | | | | |
|---|---|---|---|---|---|---|---|---|---|---|---|---|
| $p$ | $c$ | $p_{FWE-corr}$ | $q_{FDR-corr}$ | $k_E$ | $p_{uncorr}$ | $p_{FWE-corr}$ | $q_{FDR-corr}$ | $F$ | $(Z_E)$ | mm | mm | mm |
| 0.000 | 8 | 0.000 | 0.142 | 317 | 0.055 | 0.000 | 0.000 | 63.73 | 7.17 | 17 | -34 | -5 |
| – | – | 0.000 | 0.000 | 2453 | 0.000 | 0.000 | 0.000 | 63.68 | 7.17 | 27 | -40 | -35 |
| – | – | – | – | – | – | 0.000 | 0.009 | 43.75 | 6.07 | 15 | -41 | -43 |
| – | – | 0.000 | 0.092 | 431 | 0.028 | 0.000 | 0.001 | 52.51 | 6.59 | -13 | -63 | -26 |
| – | – | 0.000 | 0.088 | 491 | 0.020 | 0.000 | 0.001 | 52.40 | 6.58 | 15 | -60 | -31 |
| – | – | 0.000 | 0.003 | 1404 | 0.000 | 0.000 | 0.001 | 52.34 | 6.58 | -14 | -42 | -41 |
| – | – | – | – | – | – | 0.000 | 0.004 | 46.15 | 6.22 | -27 | -43 | -38 |
| – | – | 0.000 | 0.352 | 143 | 0.181 | 0.000 | 0.004 | 47.28 | 6.29 | -16 | -35 | -3 |
| – | – | 0.000 | 0.361 | 118 | 0.222 | 0.000 | 0.032 | 40.03 | 5.83 | 2 | -68 | -16 |
| – | – | 0.000 | 0.352 | 137 | 0.190 | 0.000 | 0.198 | 34.64 | 5.45 | 25 | -89 | -37 |
