# Supplementary material for: Tbx1 haploinsufficiency leads to local skull deformity, paraflocculus and flocculus dysplasia, and motor-learning deficit in 22q11.2 deletion syndrome
Source: Nat Commun. 2024 Dec 5;15:10510. doi: 10.1038/s41467-024-54837-3 (PMC11621701; doi:10.1038/s41467-024-54837-3)
Supplement: Supplementary file 2 — Description of Additional Supplementary Files [file 41467_2024_54837_MOESM2_ESM.pdf]

Description of Additional Supplementary Information

Supplementary Movies 1, 2. A representative 3D rendering of the surfaces resulting from the parcellation of ex vivo MRI data from a 2-month-old WT brain (Supplementary Movie 1) and a 2- month-old Df(16)1/+ brain (Supplementary Movie 2). Transparent red, cortex; light green, hippocampus; dark green, thalamus; magenta, cerebellum. Supplementary Movie 2 shows a defect in PF/F development in Df(16)1/+ mice.

Supplementary Movies 3, 4. A representative 3D rendering resulting from the parcellation of combined highresolution CT and ex vivo MRI data concurrently collected in a WT mouse (Supplementary Movie 3) and a Df(16)1/+ mouse (Supplementary Movie 4). Grey, CT data, cranium; orange, MRI data, brain. Data show the PF/F (bright orange) occupancy inside the SF cavity. Supplementary Movie 4 shows the limited occupancy of the PF/F within the deformed SF in a Df(16)1/+ mouse.
