# Supplementary material for: Tbx1 haploinsufficiency leads to local skull deformity, paraflocculus and flocculus dysplasia, and motor-learning deficit in 22q11.2 deletion syndrome
Source: Nat Commun. 2024 Dec 5;15:10510. doi: 10.1038/s41467-024-54837-3 (PMC11621701; doi:10.1038/s41467-024-54837-3)
Supplement: Supplementary file 7 — Reporting Summary [file 41467_2024_54837_MOESM7_ESM.pdf]

nature portfolio

Corresponding author(s): Zakharenko, Stanislav S.

Last updated by author(s): Sep 23, 2024

Reporting Summary

Nature Portfolio wishes to improve the reproducibility of the work that we publish. This form provides structure for consistency and transparency in reporting. For further information on Nature Portfolio policies, see our Editorial Policies and the Editorial Policy Checklist.

Statistics

For all statistical analyses, confirm that the following items are present in the figure legend, table legend, main text, or Methods section.

n/a	Confirmed	
☐	☒	The exact sample size (*n*) for each experimental group/condition, given as a discrete number and unit of measurement
☐	☒	A statement on whether measurements were taken from distinct samples or whether the same sample was measured repeatedly
☐	☒	The statistical test(s) used AND whether they are one- or two-sided<br>*Only common tests should be described solely by name; describe more complex techniques in the Methods section.*
☐	☒	A description of all covariates tested
☐	☒	A description of any assumptions or corrections, such as tests of normality and adjustment for multiple comparisons
☐	☒	A full description of the statistical parameters including central tendency (e.g. means) or other basic estimates (e.g. regression coefficient) AND variation (e.g. standard deviation) or associated estimates of uncertainty (e.g. confidence intervals)
☐	☒	For null hypothesis testing, the test statistic (e.g. *F*, *t*, *r*) with confidence intervals, effect sizes, degrees of freedom and *P* value noted<br>*Give P values as exact values whenever suitable.*
☒	☐	For Bayesian analysis, information on the choice of priors and Markov chain Monte Carlo settings
☒	☐	For hierarchical and complex designs, identification of the appropriate level for tests and full reporting of outcomes
☐	☒	Estimates of effect sizes (e.g. Cohen's *d*, Pearson's *r*), indicating how they were calculated

Our web collection on statistics for biologists contains articles on many of the points above.

Software and code

Policy information about availability of computer code

Data collection	To acquire proprietary human MRI data, Siemens software was used. Basic demographic data were stored on a RedCap server. The animal MRI study was performed using a 7T Bruker ClinScan system (Bruker BioSpin MRI GmbH, Ettlingen, Germany) equipped with a 12S-gradient coil. In CT experiments, mouse skulls were scanned using the Inveon PET/CT system (Siemens), at 14-µm resolution for the high-resolution scans and 45-µm resolution for the low-resolution scans. Alternatively, mice were scanned on a Bruker Skyscan 1276 CT (Bruker Biospin) at 18.59-µm resolution, at 70 kV and 200-µA and using a 0.5-mm aluminum filter. Whole-cell current-clamp recordings were made with patch pipettes (3-5 MΩ) by using a Multiclamp 700B amplifier, digitized (10 kHz) with a Digidata 1440, and recorded using pCLAMP 10 software (all Molecular Devices, San Jose, CA). Histological analysis, immunohistochemistry, and neuronal migration assays were performed using LSM780 (ZeissAxioObserver). Data for the VOR experiments were collected using the eye-tracking software (ETL-200). The snRNA-seq sequencing was performed using 10× Genomics (Pleasanton, CA). Libraries were sequenced on an Illumina NovaSeq6000 Sequencing System (Illumina, San Diego, CA).

Data analysis	Mango version 4.1, MAGeT, Statistical Parametric Mapping software package (SPM12), MATLAB® (vR2020a), Prism10, OsiriX (v5.7), Inveon Research Workplace software (IRW 4.2), 3D Slicer, CIVET pipeline (v2-1-1), SoupX software (v1.6.2), clusterProfiler package (v4.6.2), Cell Ranger software (v7.0.0)M MSigDB (v7.4.1), DCATS (v1.1.0), R (v4.3.1).<br><br>The code used for the analysis of snRNA-seq data is available at Github: https://github.com/ZakharenkoLab/Tbx1_haploinsufficiency_snRNAseq_Project. Code to perform MAGeT cerebellar volumetric segmentation is public and freely available at https://github.com/CobraLab/MAGeTbrain. The SUIT toolbox used to perform voxel-based morphometry is similarly publicly available and can be accessed at https://www.diedrichsenlab.org/imaging/suit.htm. Both websites also provide detailed instructions on how to implement these tools on standard MR datasets.

For manuscripts utilizing custom algorithms or software that are central to the research but not yet described in published literature, software must be made available to editors and reviewers. We strongly encourage code deposition in a community repository (e.g. GitHub). See the Nature Portfolio guidelines for submitting code & software for further information.

Data

Policy information about availability of data

All manuscripts must include a data availability statement. This statement should provide the following information, where applicable:

- Accession codes, unique identifiers, or web links for publicly available datasets
- A description of any restrictions on data availability
- For clinical datasets or third party data, please ensure that the statement adheres to our policy

All relevant data associated with the published study are present in the paper or the Supplementary Information. The source data underlying the main and supplementary figures are provided as a Source Data file. The RNA-seq data generated in this study are available in the NCBI Gene Expression Omnibus database under accession code GSE254044 [https://www.ncbi.nlm.nih.gov/geo/query/acc.cgi?acc=GSE254044]. The 22q11.2 human subjects data used in this and previous studies are not publicly available. However, they are included as part of the ENIGMA-22 Consortium dataset, which is available to qualified investigators. Neuroimaging data from the Philadelphia Neurodevelopmental Cohort are publicly available and can be accessed through dbGAP at https://www.ncbi.nlm.nih.gov/projects/gap/cgi-bin/study.cgi?study_id=phs000607.v1.p1. Additional data relating to this paper are available upon request from the corresponding author, because the size (>5 TB) of the MRI and CT imaging, immunochemistry, animal behavior, and electrophysiology data is too large to be deposited online. Source Data are provided with this paper.

Research involving human participants, their data, or biological material

Policy information about studies with human participants or human data. See also policy information about sex, gender (identity/presentation), and sexual orientation and race, ethnicity and racism.

Reporting on sex and gender	Sex was determined via self-report and confirmed with genotyping. Sex/gender-specific effects on the PF/F volumes were analyzed, and no difference between sexes was found; this result is described in Supplementary Fig. 2d. Thus, both sexes were included in the rest of our analyses. Of the subjects with 22q11DS, 47.5% were male and 62% of typically developing (TD) controls were male. Consent has not been obtained for sharing individual demographic information.
Reporting on race, ethnicity, or other socially relevant groupings	N/A
Population characteristics	Both age and sex are predicted to have an effect on brain measures, particularly volumetric measures. Therefore, we performed analyses that included these measures as covariates.
Recruitment	Subjects with 22q11DS were recruited through the 22q and You Center at the Children's Hospital of Philadelphia (CHOP), one of the largest clinics of its kind in the world. The Center treats a broad spectrum of individuals with the 22q11.2 deletion, and its population is relatively unbiased. However, the exclusion criteria used (e.g., IQ >70) could potentially bias the sample towards higher-functioning individuals; this exclusion was performed primarily to ensure high-quality imaging data.<br><br>The TD controls were subsampled from the Philadelphia Neurodevelopmental Cohort (PNC) and matched for demographic characteristics of the 22q11DS sample. PNC subjects were recruited from a prospective population-based study by the Center for Applied Genomics (CAG) at CHOP, as part of a study investigating the genetics of complex traits in ~10,000 youths. The original CAG recruitment was based on individuals who visited a clinician in the CHOP network for a pediatric visit. The PNC imaging subjects were, in turn, selected from this cohort via random subsampling.
Ethics oversight	The Human Subjects portion of the study was approved by both the Penn and CHOP IRB.

Note that full information on the approval of the study protocol must also be provided in the manuscript.

Field-specific reporting

Please select the one below that is the best fit for your research. If you are not sure, read the appropriate sections before making your selection.

☒ Life sciences ☐ Behavioural & social sciences ☐ Ecological, evolutionary & environmental sciences

For a reference copy of the document with all sections, see nature.com/documents/nr-reporting-summary-flat.pdf

Life sciences study design

All studies must disclose on these points even when the disclosure is negative.

Sample size	No sample-size calculation was performed. Sample size was determined based on our experience and the sample size used in similar studies. MRI data for rodent studies were from 3-21 mice; MRI data for the human study were from TD controls (n=68) and subjects with 22q11DS (n=80); VOR data were from 5-7 mice; LTP/LTD study data were from 4-5 mice; immunohistochemistry data for cerebellar cell compositions and neurogenesis were from 2-6 mice; CT data were from 4-16 mice; snRNA-seq data were from 10 mice per group.<br>Human MRI studies: the 22q11DS sample included all available subjects from prior study and a comparable-sized control group. Specific power calculations for floccular and parafloccular analysis were not performed. Prior studies of this sample have found significant differences in brain morphology between TD controls and subjects with 22q11DS. However, our analysis showed a much smaller effect than was expected based on preliminary results in the mouse model.
Data exclusions	No data were excluded from the analysis; we could not obtain data from 1-2 mice during the behavior test because they closed their eyes during the test. Thus, those mice had to be omitted from data collection in Fig. 3f, g.<br>Human MRI: No data were excluded from the analyses. However, there were prospective exclusion criteria to determine the imaging subsample in the 22q11DS group (i.e., IQ > 70, no neurological disorders), which biased the sample toward. higher-functioning individuals. Therefore, these results may understate group differences in 22q11DS, relative to an unselected 22q11DS sample.
Replication	All ex vivo and in vivo experiments were repeated by separate grouping analysis. For example, snRNA-seq data were measured in two separate batches (n=5 per batch).<br>Human MRI: Due to the rarity of 22q11DS, difficulty in obtaining high-quality data, and computational demands of some analyses, replication with an independent sample was not performed. However, the study used several different analytic apporaches to demonstrate that the findings were not method-specific, and the findings in the human subjects support similar findings in the mouse models.
Randomization	Mice in groups were randomly assigned to different experimental conditions.<br>Human MRI: the presence of the 22q11DS deletion defined the experimental sample.
Blinding	Mice data: all investigators were blined to group allocation during data collection and analysis.<br>Human MRI: During manual tracing of floccular ROIs, the rater was blinded to diagnosis. Investigators were not blinded during data collection. However, the MRI protocols used were standardized across all subjects and would not be affected by knowledge of diagnosis. For VBM analysis, diagnosis was known (to specify group contrasts), but the process is entirely data-driven, highly automated, and not particularly prone to biases related to the lack of blinding.

Reporting for specific materials, systems and methods

We require information from authors about some types of materials, experimental systems and methods used in many studies. Here, indicate whether each material, system or method listed is relevant to your study. If you are not sure if a list item applies to your research, read the appropriate section before selecting a response.

Materials & experimental systems

n/a	Involved in the study	
☐	☒	Antibodies
☒	☐	Eukaryotic cell lines
☒	☐	Palaeontology and archaeology
☐	☒	Animals and other organisms
☒	☐	Clinical data
☒	☐	Dual use research of concern
☒	☐	Plants

Methods

n/a	Involved in the study	
☒	☐	ChIP-seq
☒	☐	Flow cytometry
☐	☒	MRI-based neuroimaging

Antibodies

Antibodies used	Rabbit anti–cleaved caspase-3, 1:250, Cell Signaling Technology, Cat#9661S, RRID: AB_2341188<br>Mouse anti-PH3, 1:100, Santa Cruz Biotech, Cat#sc-374669, RRID: AB_11150094<br>Rabbit anti-Ki67, 1:500, Abcam Cat#ab15580, RRID: AB_443209<br>Rabbit anti–calbindin D-28K, 1:1000, Millipore Sigma, Cat#ABN2192, RRID: AB_2935805<br>Mouse anti-PAX6, 1:5, DSHB, Cat#pax6; RRID: AB_528427<br>Rabbit anti-GFAP, 1:250, DAKO Cat#Z0334, RRID: AB_10013382<br>Rabbit anti-Tbr2, 1:500, Abcam, Cat#ab23345, RRID: AB_778267<br>Mouse anti–beta III-tubulin, 1:500, Sigma, Cat#T8660; RRID: AB_477590<br>Goat anti–rabbit IgG, Alexa-546, 1:1000, ThermoFisher Scientific Cat#A11071, RRID: AB_1500774<br>Goat anti–mouse IgG, Alexa-488, 1:1000, ThermoFisher Scientific Cat#A28175,RRID: AB_2536161
Validation	All antibodies applied were previously validated for use in multiple species by multiple studies (see company websites for details).

Animals and other research organisms

Policy information about studies involving animals; ARRIVE guidelines recommended for reporting animal research, and Sex and Gender in Research

Laboratory animals	All mouse strains were backcrossed onto the C57/BL6 genetic background (Charles River Laboratories) and both male and female mice were used from P3 to 8 months old.<br>Mouse: Del(3.0 Mb)/+, Saito et al., 2020, RRID: IMSR _RBRC11066<br>Mouse: LgDel/+, Merscher et al., 2001, RRID: IMSR_MGI:3702642<br>Mouse: Df(16)1/+, Lindsay et al., 1999, RRID: IMSR_MGI:3623881<br>Mouse: Znf74l-Ctp/+, Kimber et al., 1999, RRID: IMSR_MGI:3701447<br>Mouse: Rtn4r+/– , Kim et al., 2004, RRID: IMSR_MGI:3514018<br>Mouse: Comt+/–, Gogos et al., 1998, RRID: IMSR_MGI:88470<br>Mouse: T10+/–, generated in this paper<br>Mouse: Gnb1l+/–, Paylor et al., 2006, RRID: IMSR_MGI:1338057<br>Mouse: Df(16)2/+, Lindsay et al., 2001<br>Mouse: Df(16)3/+, Lindsay et al., 2001<br>Mouse: Df(16)4/+, Lindsay et al., 2001<br>Mouse: Df(16)5/+, Lindsay et al., 2001<br>Mouse: Dgcr8+/–, Lindsay et al., 2001<br>Mouse: Tbx1+/–, Lindsay et al., 2001<br>Mouse: Sept5+/–, Lindsay et al., 2001, RRID: IMSR_MGI:3701447<br>Mouse: Arvcf-Txnrd2/+, generated in this paper<br>Mouse: Tbx1fl/+, Arnold et al., 2006, RRID: IMSR_MGI:3701447<br>Mouse: Tbx1-GFPfl/+, Freyer et al., 2013, RRID: IMSR_JAX:037516<br>Mouse: Mesp1Cre, Saga et al., 1999, RRID: IMSR_ MGI:2176467<br>Mouse: Tie2Cre, Kisanuki et al., 2001, RRID: IMSR_JAX:008863<br>Mouse: NeuroD1Cre, Gong et al., 2007, RRID: IMSR_ JAX:028364<br>Mouse: L7Cre, Oberdick et al., 1990, RRID: IMSR_ JAX:004146<br>Mouse: Twist2Cre, Šošić et al., 2003, RRID: IMSR_ JAX:008712<br>Mouse: Lyz2Cre, Clausen et al., 1999, RRID: IMSR_ JAX:018956<br>Mouse: Foxg1Cre, Hébert et al., 2000, RRID: IMSR_ JAX:006084<br>Mouse: p53+/–, Jacks et al., 1994, RRID: IMSR_ JAX:002101
Wild animals	This study did not involve wild animals.
Reporting on sex	A sex-based test was performed, and the result is described in Supplementary Fig. 1a. As no difference between sexes was found, we applied both sexes for the rest of the analyses.
Field-collected samples	This study did not involve samples collected from the field.
Ethics oversight	The care and use of animals were reviewed and approved by the St Jude Children's Research Hospital Institutional Animal Care and Use Committee, in accordance with US National Institutes of Health (NIH) guidelines on Care and Use of Laboratory Animals.

Note that full information on the approval of the study protocol must also be provided in the manuscript.

Plants

Seed stocks	N/A
Novel plant genotypes	N/A
Authentication	N/A

Magnetic resonance imaging

Experimental design

Design type	Structural MRI, case-control design
Design specifications	N/A
Behavioral performance measures	N/A

Acquisition

Imaging type(s)	Structural MRI
Field strength	7T for mice; 3 Tesla for human
Sequence & imaging parameters	Mice: T2-weighted turbo spin-echo sequence (Repetition time/Echo time=3841/50ms, field of view=25×25mm, matrix =320×320 pixels, echo train length=7, number of slices=42, number of averages=1, thickness=0.4mm, scan time=6.5 min).<br>Human: Sagittal MP-RAGE.<br>FOV=180x240<br>Matrix size 192x256x160<br>Slice Thickness 1 mm<br>TR/TE 1810/3.51ms<br>Flip Angle: 9 degrees
Area of acquisition	Mice: whole-brain scan was performed, followed by volume analysis of whole brain, cerebellum, paraflocculus/ flocculus, CrusI/II, Vermis IV/V.<br>Human: whole-brain scans were performed, but analyses were limited to the cerebellum.

Diffusion MRI ☐ Used ☒ Not used

Preprocessing

Preprocessing software	Human: *Floccular ROIs were manually measured on native MRI scans in Mango.<br>*Other cerebellar volumetric measures were preprocessed through CIVET with both affine and nonaffine registration<br>*VBM analyses were analyzed using the standard SPM12 pipeline
Normalization	Human: *Floccular ROIs were manually measured on native data, comparable to clinical reads.<br>*Other cerebellar volumetric measures were processed through CIVET using N3 intensity normalization<br>*VBM analyses were normalized using the Dartel algorithm in SUIT
Normalization template	Human: *Floccular ROIs were manually measured without standardizing to a template.<br>*Data for other cerebellar volumetric measures were first normalized to the MNI ICBM152 model via the CIVET pipeline. Cerebellar parcellation was subsequently based on a multiatlas template, as part of the MAGeT segmentation algorithm.<br>*VBM analyses were performed using the standard SUIT atlas cerebellar template, which is based on the ICBM152 Template but provides greater anatomic detail of the cerebellum.
Noise and artifact removal	Human: *Floccular ROIs were manually measured on raw MRI data.<br>*Other cerebellar volumetric measures used standard CIVET QC pipelines.<br>*Preprocessing for VBM used standard structural SPM QC pipelines.
Volume censoring	N/A

Statistical modeling & inference

Model type and settings	Human: *Analysis of both manual floccular ROIs and cerebellar volumes used serial univariate analyses. Both linear regression models and t-tests were performed (to improve comparisons with mouse results), resulting in similar statistical inferences.<br>*VBM analysis is an effective, massively univariate voxel-level ANOVA, with control of multiple testing performed both via selection of a family-wise error threshold (p-value = 0.0001) and a minimal cluster size of voxels below the probabilistic threshold. All tests were two-tailed.
Effect(s) tested	All statistical tests for the human subjects component of the study evaluated 22q11DS vs. TD group differences

Specify type of analysis: ☐ Whole brain ☒ ROI-based ☐ Both

Anatomical location(s)	Human: *Flocculus measures were determined based on expert tracings.<br>*Other volumetric measures were based on automatic parcellation with MAGeT and visually inspected for accuracy.<br>*VBM analysis was probabilistic, though the analysis was restricted to the automatically generated SUIT cerebellar template (in other words, no whole-brain VBM).
Statistic type for inference<br>(See Eklund et al. 2016)	For VBM analysis, SPM12 uses both voxel-wise and cluster-wise statistics, based on the assumptions of Gaussian random field theory. Data were smoothed using an 8-mm kernel prior to statistical modeling.
Correction	VBM used an FWE threshold of p-value = 0.001 and a cluster threshold of k = 100.

Models & analysis

n/a	Involved in the study
☒	☐ Functional and/or effective connectivity
☒	☐ Graph analysis
☒	☐ Multivariate modeling or predictive analysis
